# Supplementary material for: Uncovering 2-D toroidal representations in grid cell ensemble activity during 1-D behavior
Source: Nat Commun. 2024 Jun 26;15:5429. doi: 10.1038/s41467-024-49703-1 (PMC11208534; doi:10.1038/s41467-024-49703-1)
Supplement: Supplementary file 1 — Supplementary Information [file 41467_2024_49703_MOESM1_ESM.pdf]

Supplementary Information

Uncovering 2-D toroidal representations in grid cell ensemble activity during 1-D behavior

Supplementary Figures

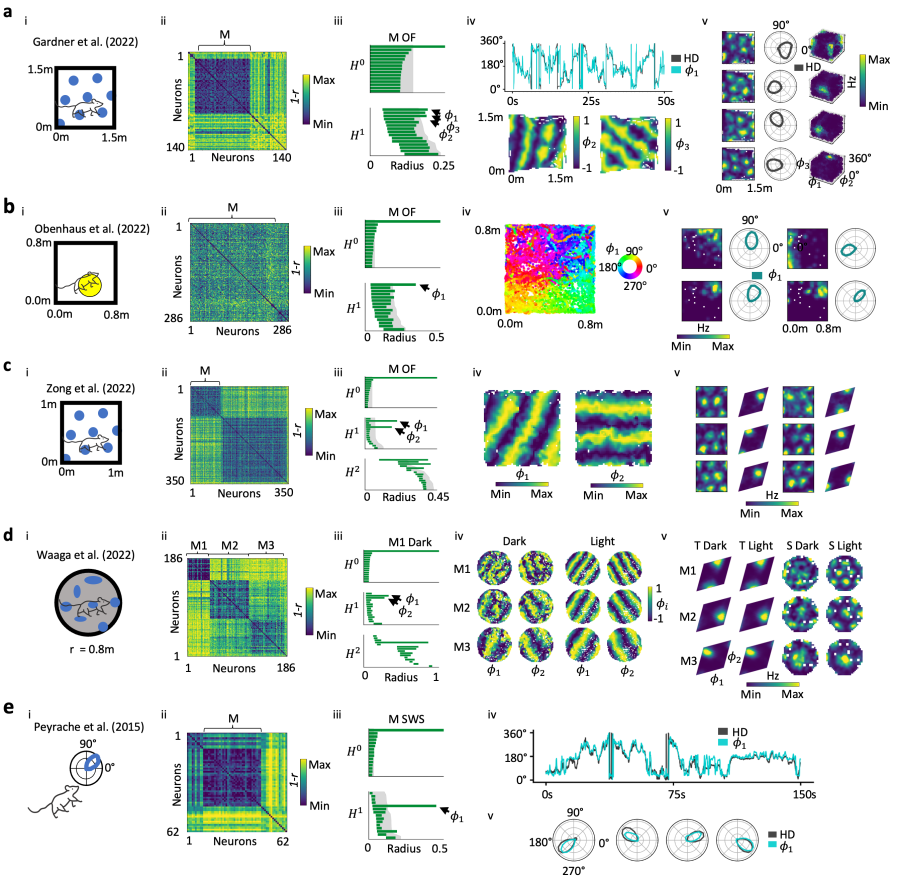

Supplementary Fig. 1 Finding neural representations in under different experimental conditions. **a.** Grid cell population analysis as in Fig. 1 for rat 'S' [1]. i) Illustrative grid cell spatial tuning. ii) Correlation distance matrix. Ensemble ('M', $n = 78$ cells) indicated. iii) Barcode diagram. Arrows indicating prominent circular features (ϕ_{1-3}). iv) 50 s snippet of decoded circular feature and the recorded head direction (top), and the mean cosine values of the two remaining decoded features as a function of space. v) Single-cell tuning of four neurons to recorded position (left), head direction (middle) and decoded 3-D torus (right). **b.** Ring topology detected in ensemble activity ('M', $n = 169$ cells) during OF exploration (as in Fig. 2, data from Obenhaus et al. [2]) corresponding to boundary vector cell representation (i). ii-iv) as in **a**. v) Single-cell tuning to physical space (left in each pair) and to decoded circular feature (right). **c.** Toroidal topology of entorhinal ensemble activity ('M', $n = 109$ cells) of calcium recordings during open field [3], Similar to Fig. 2. ii-v) as in **a**, for 2-D torus. **d.** Toroidal topology of grid cell ensemble activity (three detected ensembles: 'M1', $n = 42$ cells; 'M2', 73 cells; 'M3', 65 cells) in both dark and light sessions (i, data from Waaga et al. [4]). ii-v) as in **c**. **e.** Ring topology in ensemble activity ($n = 36$ cells) from electrophysiological recordings in ADn and postsubiculum during SWS corresponding to head direction circle (i, data from Peyrache et al. [5]). ii,iii) as in **a**. iv) 150s snippet of the decoded circular coordinates (using the parameterization during SWS to decode OF session data) and the recorded head direction. v) Single-cell tuning to the circular feature found during SWS and awake head direction for four example cells.

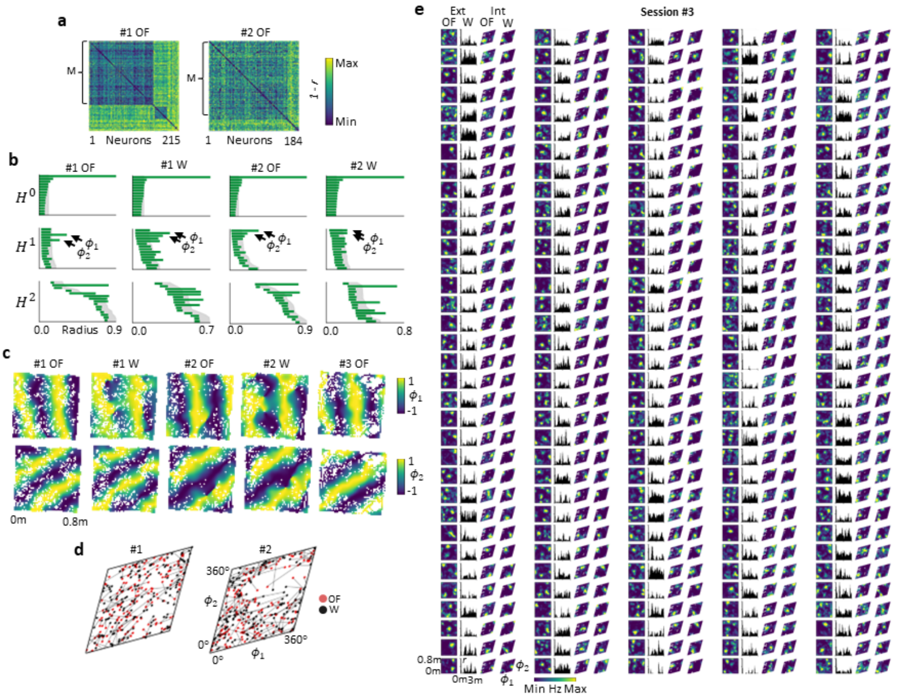

Supplementary Fig. 2 Barcodes, spatial mappings, distance matrices and single-cell responses for calcium recordings not shown in Fig. 2. **a.** Correlation distance matrices for two open field recording days (1 and 2) of same mouse as in Fig. 2b. The clusters analyzed are marked as 'M' (n = 154 and 161 cells). **b.** Barcodes (as in Fig. 2c). **c.** Mean toroidal coordinates as a function of OF locations (as in Fig. 2d). For W sessions, OF toroidal coordinates are obtained by using the toroidal parametrization found during wheel running to decode the activity during the OF session. Note, the toroidal axes are reoriented to match across sessions. **d.** Distributions of toroidal receptive field centers (as in Fig. 2f) for each cell across W and OF environments. **e.** Tuning to position on physical space ('Ext') and on the inferred torus ('Int') for ensemble found for session #3 (five of which are shown in Fig. 2e).

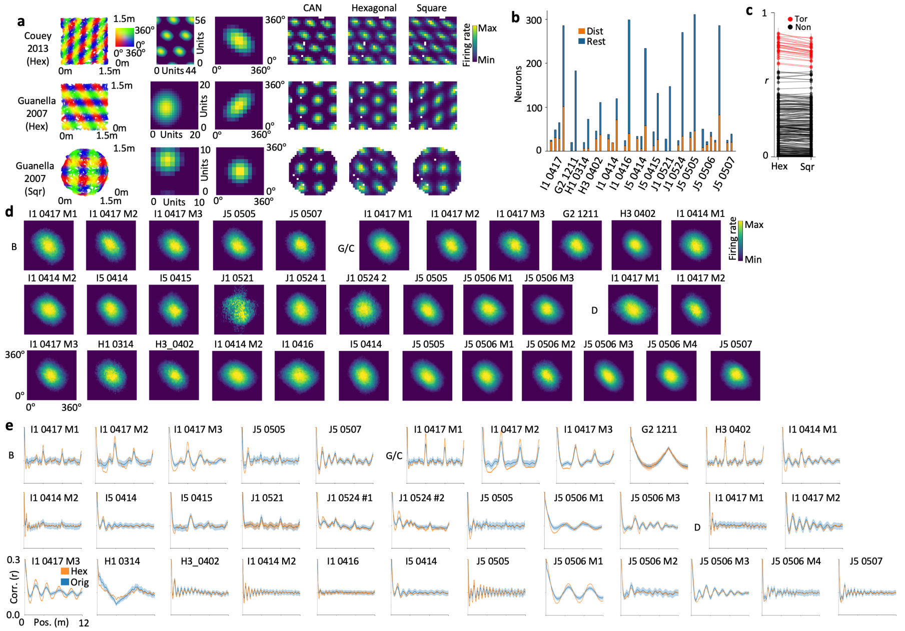

Supplementary Fig. 3 Decoding of toroidal coordinates in simulated grid cell and entorhinal ensemble activity allows classifying toroidal ensembles and comparing square and hexagonal toroidal structure. **a.** Toroidal coordinates from topological analyses on data generated by three CAN models (row-wise, from top): an inhibitory, multi-bump network model, a twisted and an un-twisted (square) torus network model ($n = 2464, 400$ and 100 units, respectively). Each row displays (from left): a 2-D spatial trajectory colored according to toroidal position (2-D color map); firing rates of each neuron at a chosen time frame, ordered according to network connectivity (firing rate given by color bar); stacked, centered toroidal rate maps of all neurons in the network; spatial rate maps of activity from a CAN grid cell and fitted data using hexagonal and square torus point source models. Note angle of the stacked rate maps. **b.** Bar plot of number of neurons in the analysis for each toroidal ensemble (left, rightmost bar showing whole population per session) classified by hexagonal toroidal rate map classification of each cluster (as in Fig. 3b,e and Supplementary Fig. 4a). Orange showing number of 'distance' cells classified by autocorrelagrom as in Campbell et al. [6] (number of cells classified as distance/number of cells in total, mouse and day, from left: I1 0417, 23/25 (M1), 30/49 (M2), 29/65 (M3), 101/286 (all); G2 1211, 0/20 (M1), 0/183 (all); H1 0314, 6/20 (M1), 6/73 (all); H3 0402, 28/46 (M1), 37/111 (all); I1 0414, 19/19 (M1), 28/38 (M2), 71/120 (all); I1 0416, 12/24 (M1), 40/299 (all); I5 0414, 29/34 (M1), 5/34 (M2), 58/234 (all), I5 0415, 12/44 (M1), 26/132 (all); J1 0521 0/28 (M1), 0/147 (all); J1 0524, 14/24 (M1), 34/270 (all); J5 0505, 31/43 (M1), 46/311 (all); J5 0506, 8/50 (M1), 15/22 (M2), 34/43 (M3), 21/25 (M4) and J5 0507, 19/25 (M1), 20/39 (all). **c.** Median toroidal scores (hexagonal vs square torus, ± S.E.M.) based on the parametrization of the two longest-lived circular features found for each cluster ($n = 119$ clusters) with more than 18 cells across all 119 MEC sessions. Ensembles with median value above 0.6 were characterized as toroidal (red). **d.** Stacked, centered toroidal rate maps of all neurons in each ensemble (n as in **b**), visually oriented across ensembles. Note similarity with hexagonal CANs in **a**. **e.** Mean spatial autocorrelograms (± s.e.m) for each toroidal ensemble (as in **d**) for recorded data (blue) and data generated by the hexagonal torus model (orange).

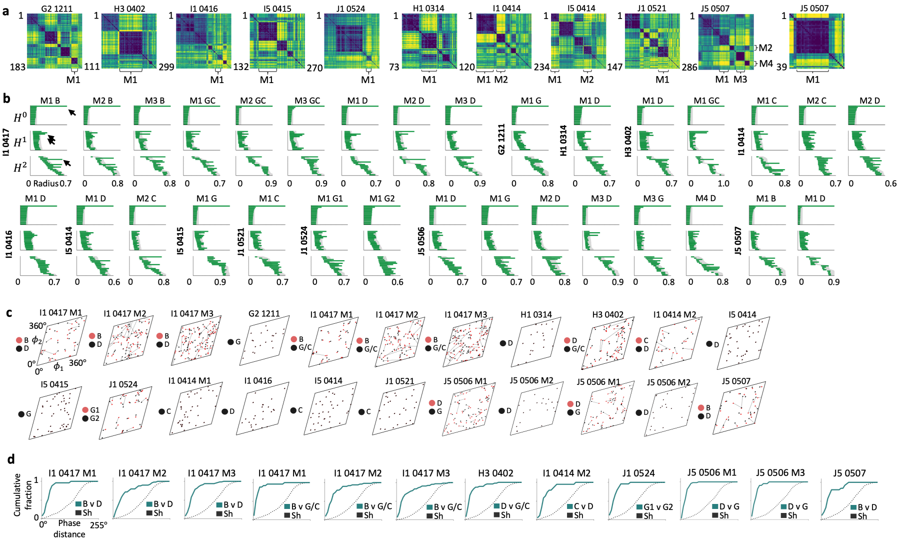

Supplementary Fig. 4 Correlation distance matrices, barcodes and toroidal phase statistics for ensembles classified as toroidal, not shown in Fig. 3. **a.** Correlation distance matrices (as in Fig. 3b) sorted by cluster indices. The clusters analyzed are marked as 'M*' ($n = 19-65$ cells, as in Supplementary Fig. 3b). **b.** Barcode diagrams (as in Fig. 3c) for each ensemble and session. **c, d.** Distributions of receptive field centers on inferred torus and cumulative distributions of phase distances compared to shuffle (as in Fig. 3f) for all neurons in each ensemble, compared across sessions.

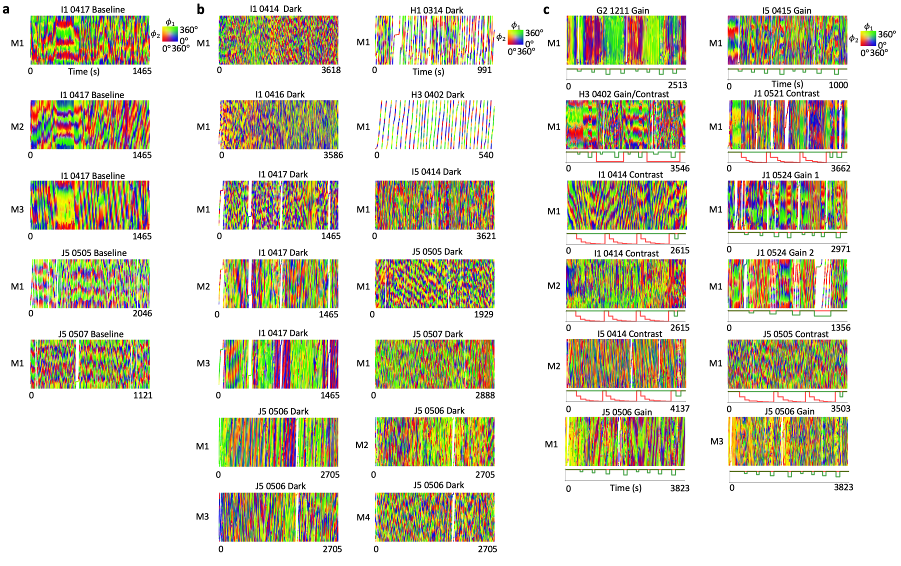

Supplementary Fig. 5 Internal toroidal dynamics as a function of the VR track for sessions not shown in Fig. 3g. **a-c.** Spatio-temporal positions colored by the 2-D toroidal positions across the entire session for baseline, gain, contrast and dark sessions. Gain and contrast manipulations are indicated in green and red line plots below relevant sessions (c). Note, in dark, the mice cannot see a progression of VR linear track.

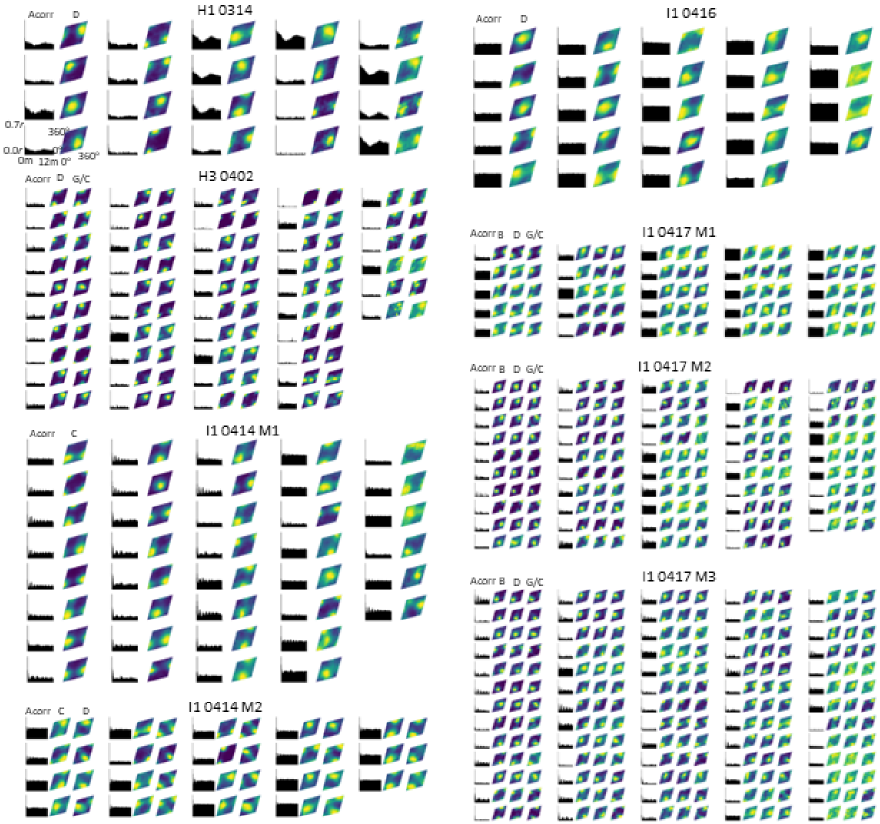

Supplementary Fig. 6 Tuning to coordinates in space and on the inferred torus for all neurons of toroidal ensemble(s) of mouse and experimental day: H1 0314 (20 neurons), H3 0402 (46 neurons), I1 0414 M1 (38 neurons) and M2 (19 neurons), I1 0416 (24 neurons) and I1 0417 M1 (25 neurons), M2 (49 neurons) and M3 (65 neurons). Plots from left to right: 1-D VR-track autocorrelogram during dark session, toroidal firing rate map for baseline (B), gain (G), contrast (C) or dark (D)

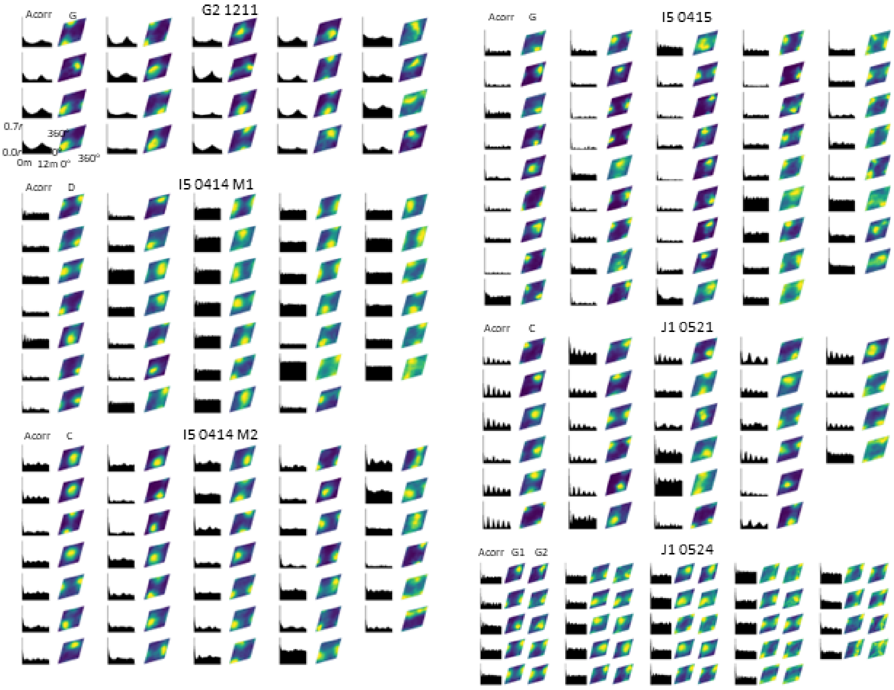

Supplementary Fig. 7 Tuning to coordinates in space and on the inferred torus for all neurons of toroidal ensemble(s) of mouse and experimental day: G2 1211 (20 neurons), I5 0414 M1 (34 neurons) and M2 (34 neurons), I5 0415 (44 neurons), J1 0521 (28 neurons) and J1 0524 (24 neurons). Plots from left to right: 1-D VR-track autocorrelogram during dark session, toroidal firing rate map for baseline (B), gain (G), contrast (C) or dark (D)

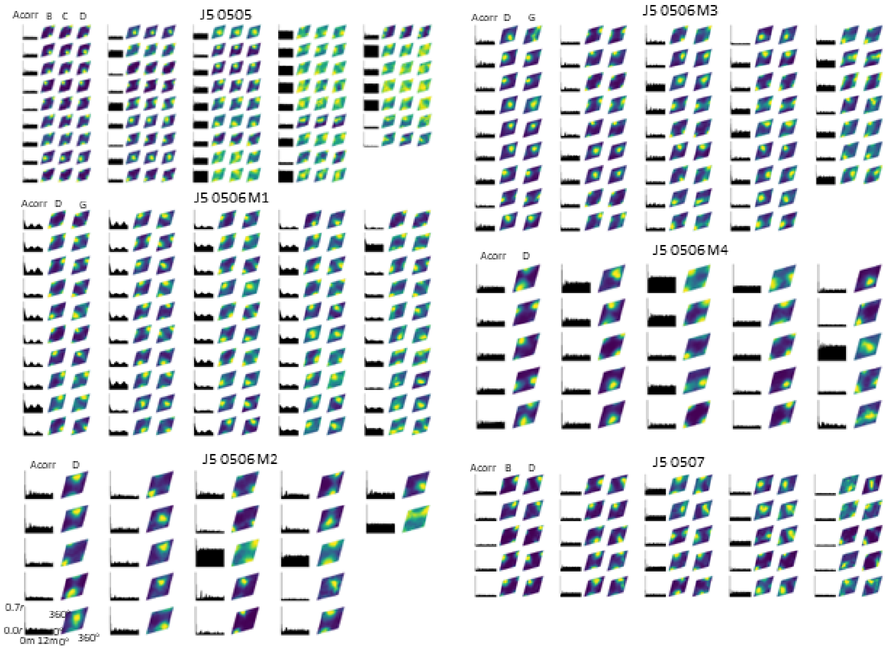

Supplementary Fig. 8 Tuning to coordinates in space and on the inferred torus for all neurons of ensembles of mouse J5, experimental day: 0505 (43 neurons), 0506 M1 (50 neurons), M2 (22 neurons), M3 (44 neurons) and M4 (25 neurons) and 0507 (25 neurons). Plots from left to right: 1-D VR-track autocorrelogram during dark session, toroidal firing rate map for baseline (B), gain (G), contrast (C) or dark (D)

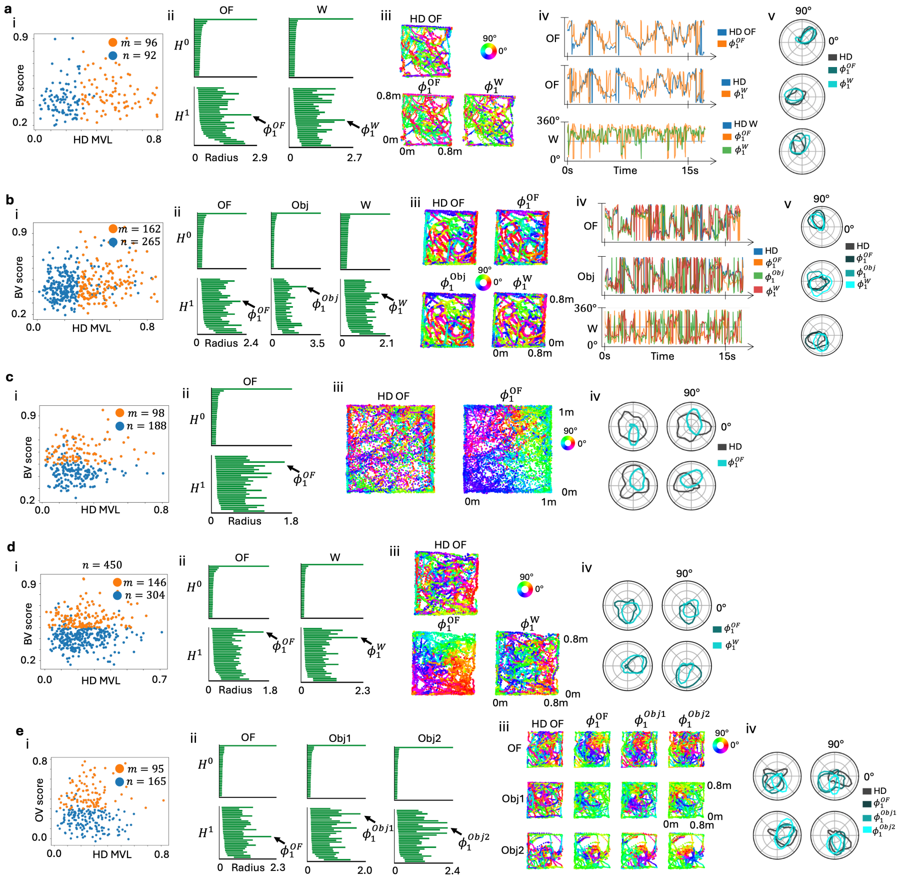

Supplementary Fig. 9 Examples of preliminary exploration of other MEC cell types found in calcium imaging data from Obenhaus et al. [2]. **a,b.** Ring topology of head direction (HD) cell network in Mouse 82913 (Metasessions '8b7cea64d65789d8' and 'e4cbcbf8233d1b3b', $m = 96$ and 162 cells), during open field (OF) foraging, wheel (W) running and open arena with object (Obj) exploration . i) HD mean vector length (MVL) vs. boundary vector (BV) score for all neurons (scores derived from [7]). HD cells (orange) are identified using MVL ¿ 0.3 and excluding noisy neurons. ii) Barcodes (as in Fig. 1e) with arrow indicating longest-lived 1-D feature, ϕ_1^X where X refers to experimental condition. iii) Coloring of spatial locations of the animal based on simultaneously recorded HD angle and decoded circular coordinates (as identified in ii). iv) Comparison of recorded HD angle (blue) and decoded coordinates (orange, green, red) across time. v) Single cell tuning of three example cells to recorded HD angle (black) and decoded coordinates (shades of cyan). **c,d.** Uncovering directional component of BV cell network in Mouse 90222 and 88106, Metasessions 'a936be26a1d73b28' and 'a68be582f47de42e' ($m = 98$ and 146 cells). i-iv) as in **a**, here identifying BV cells by BV score ¿ 0.5. **e.** Circular decoding of object vector (OV) cell activity in Mouse 90222, Metasession '7c8c99c304589361' ($n = 95$ cells). i-iv) as in **c,d**. Ov cells are identified by OV score ¿ 0.3.

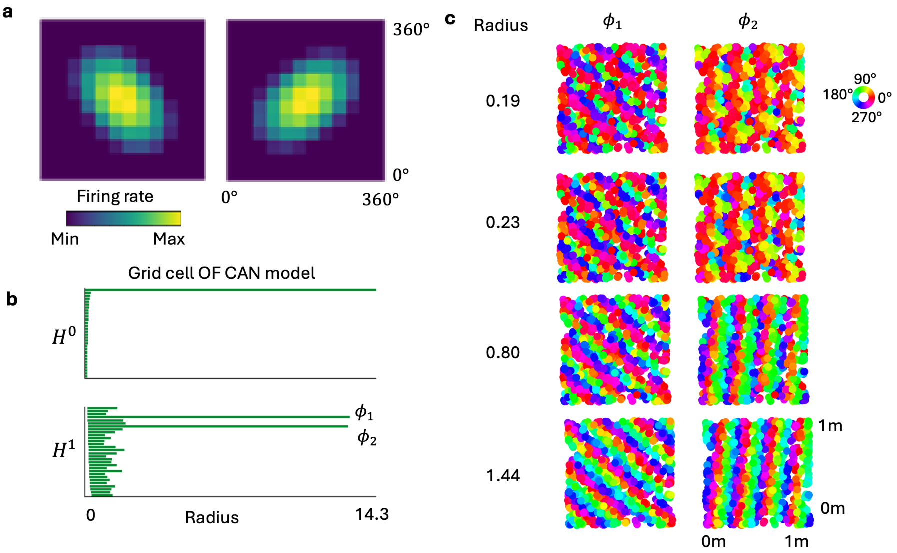

Supplementary Fig. 10 Orientation of toroidal tuning and choice of radius for coordinatization. a) The stacked, centered toroidal rate maps across a simulated grid cell module. Since the relative orientation between a pair of axes of the hexagonal torus is either 60 or 120 degrees, the activity bump in a square rate map is oriented along one of the diagonals. b) Barcode of activity from simulated grid cell CAN model in an open field arena. c) The choice of cohomology class representative may affect the coordinatization, depending on the radius for which we fix the underlying simplicial complex on which to perform the coordinatization. Coloring the spatial movement with the coordinatization of the top two H^1 bars for four low-valued radii (left-most column, compare to b) shows a distortion of the spatial association of the mapping for the two radii closest to the birth of the cohomology classes. Higher values shows the expected stripe-like patterns.

References

[1] Gardner, R.J., *et al.*: Toroidal topology of population activity in grid cells. Nature **602**(7895), 123–128 (2022)

[2] Obenhaus, H.A., *et al.*: Functional network topography of the medial entorhinal cortex. Proceedings of the National Academy of Sciences **119**(7), 2121655119 (2022)

[3] Zong, W., *et al.*: Large-scale two-photon calcium imaging in freely moving mice. Cell **185**(7), 1240–1256 (2022)

[4] Waaga, T., *et al.*: Grid-cell modules remain coordinated when neural activity is dissociated from external sensory cues. Neuron **110**(11), 1843–1856 (2022)

[5] Peyrache, A., Lacroix, M.M., Petersen, P.C., Buzsáki, G.: Internally organized mechanisms of the head direction sense. Nature neuroscience **18**(4), 569–575 (2015)

[6] Campbell, M.G., Attinger, A., Ocko, S.A., Ganguli, S., Giocomo, L.M.: Distance-tuned neurons drive specialized path integration calculations in medial entorhinal cortex. Cell reports **36**(10), 109669 (2021)

[7] Obenhaus, H., et al.: Obenhaus 2022. Archive2014 (2022). https://doi.org/10.11582/2022.00005
