# Supplementary material for: Uncovering 2-D toroidal representations in grid cell ensemble activity during 1-D behavior
Source: Nat Commun. 2024 Jun 26;15:5429. doi: 10.1038/s41467-024-49703-1 (PMC11208534; doi:10.1038/s41467-024-49703-1)
Supplement: Supplementary file 3 — Description of Additional Supplementary Files [file 41467_2024_49703_MOESM3_ESM.pdf]

**File name: Supplementary Movie 1.**
**Description: Comparison of open field foraging and toroidal population dynamics.**
Left, scatter plot of position in 2-D space of mouse 88592 day 3. Right, flattened torus, displaying toroidal population distribution at each time frame, given by first smoothing each neuron's toroidal rate map (in $50^2$ bins) spatially with a Gaussian filter with $\sigma = 3$ bins. Next, every rate map was weighted by the firing rate of the corresponding neuron at each time point and the mean value for each bin across the entire population gave the time-dependent population distribution. Bright colors indicate high population activity.

**File name: Supplementary Movie 2.**
**Description: Comparison of wheel running and toroidal population dynamics for same neural ensemble as in Supplementary Movie 1.**
Left, wheel position ($y$ axis) as a function of time ($x$ axis). Right, as in Supplementary Movie 1 for internal toroidal representation decoded during wheel running.

**File name: Supplementary Movie 3.**
**Description: Comparison of VR running and toroidal population dynamics for gain sessions of mouse J5 recording day 0505.** Left, wheel position as in Supplementary Movie 2 (top) and gain values ($y$ axis) as a function of time (bottom). Right, inferred toroidal population dynamics, as in Supplementary Movies 1 and 2, found in the given recording.
