# Supplementary material for: Uncovering 2-D toroidal representations in grid cell ensemble activity during 1-D behavior
Source: Nat Commun. 2024 Jun 26;15:5429. doi: 10.1038/s41467-024-49703-1 (PMC11208534; doi:10.1038/s41467-024-49703-1)
Supplement: Supplementary file 7 — Reporting Summary [file 41467_2024_49703_MOESM7_ESM.pdf]

nature portfolio

Corresponding author(s): Erik Hermansen
Benjamin Dunn

Last updated by author(s): Dec 15, 2023

Reporting Summary

Nature Portfolio wishes to improve the reproducibility of the work that we publish. This form provides structure for consistency and transparency in reporting. For further information on Nature Portfolio policies, see our Editorial Policies and the Editorial Policy Checklist.

Statistics

For all statistical analyses, confirm that the following items are present in the figure legend, table legend, main text, or Methods section.

n/a	Confirmed	
☐	☒	The exact sample size (*n*) for each experimental group/condition, given as a discrete number and unit of measurement
☐	☒	A statement on whether measurements were taken from distinct samples or whether the same sample was measured repeatedly
☐	☒	The statistical test(s) used AND whether they are one- or two-sided<br>*Only common tests should be described solely by name; describe more complex techniques in the Methods section.*
☐	☒	A description of all covariates tested
☒	☐	A description of any assumptions or corrections, such as tests of normality and adjustment for multiple comparisons
☐	☒	A full description of the statistical parameters including central tendency (e.g. means) or other basic estimates (e.g. regression coefficient) AND variation (e.g. standard deviation) or associated estimates of uncertainty (e.g. confidence intervals)
☐	☒	For null hypothesis testing, the test statistic (e.g. *F*, *t*, *r*) with confidence intervals, effect sizes, degrees of freedom and *P* value noted<br>*Give P values as exact values whenever suitable.*
☒	☐	For Bayesian analysis, information on the choice of priors and Markov chain Monte Carlo settings
☒	☐	For hierarchical and complex designs, identification of the appropriate level for tests and full reporting of outcomes
☐	☒	Estimates of effect sizes (e.g. Cohen's *d*, Pearson's *r*), indicating how they were calculated

Our web collection on statistics for biologists contains articles on many of the points above.

Software and code

Policy information about availability of computer code

Data collection: No software was used for data collection

Data analysis: The code from this study is available at: https://github.com/erikher/Uncovering-spatial-representations-in-large-scale-recordings/ Open-source software: Jupyter notebook 6.4.8Python version 3.9.12MySQL 5.7
Open-source Python packages:
umap 0.5.5
ripser 0.6.4
numba 0.58.1
scipy 1.11.4
numpy 1.26.2
scikit-learn 0.24.2
matplotlib 3.8.2
h5py 3.6.0
gtda 0.6.0
cv2 4.8.1
pandas 1.4.2
datajoint 0.13.5
I Python 8.2.0
Cebra 0.3.1

For manuscripts utilizing custom algorithms or software that are central to the research but not yet described in published literature, software must be made available to editors and reviewers. We strongly encourage code deposition in a community repository (e.g. GitHub). See the Nature Portfolio guidelines for submitting code & software for further information.

Data

Policy information about availability of data

All manuscripts must include a data availability statement. This statement should provide the following information, where applicable:

- Accession codes, unique identifiers, or web links for publicly available datasets
- A description of any restrictions on data availability
- For clinical datasets or third party data, please ensure that the statement adheres to our policy

Public links to all data are listed in the data availability section of the paper and copied here: https://plus.figshare.com/articles/dataset/VR_Data_Neuropixel_supporting_Distance-tuned_neurons_drive_specialized_path_integration_calculations_in_medial_entorhinal_cortex_j15041316, https://archive.sigma2.no/pages/public/datasetDetail.jsf?id=l0.11582/2022.00008, https://archive.sigma2.no/pages/public/datasetDetail.jsf?id=l0.11582/2022.00005, https://crcns.org/data-sets/thalamus/th-1, https://figshare.com/articles/dataset/foroidal_topology_of_population_activity_in_grid_cells/16764508 and https:// zenodo.org/records/6200517, https://zenodo.org/records/6200517

Research involving human participants, their data, or biological material

Policy information about studies with human participants or human data. See also policy information about sex, gender (identity/presentation), and sexual orientation and race, ethnicity and racism.

Reporting on sex and gender	This work does not involve human participants.
Reporting on race, ethnicity, or other socially relevant groupings	N/A
Population characteristics	N/A
Recruitment	N/A
Ethics oversight	N/A

Note that full information on the approval of the study protocol must also be provided in the manuscript.

Field-specific reporting

Please select the one below that is the best fit for your research. If you are not sure, read the appropriate sections before making your selection.

☒ Life sciences ☐ Behavioural & social sciences ☐ Ecological, evolutionary & environmental sciences

For a reference copy of the document with all sections, see nature.com/documents/nr-reporting-summary-flat.pdf

Life sciences study design

All studies must disclose on these points even when the disclosure is negative.

Sample size	Samples included all available cells that matched the classification criteria for the relevant cell type.
Data exclusions	Cells with very low or high firing rates (below 0.05Hz or above 10Hz) were excluded because of their unsuitability for spike/event-train analysis. Contaminated or duplicated cells were excluded, because of their unsuitability for analysis.
Replication	For the animal subjects included in the study, the main text indicates in how many recordings the effect was found. Recordings from multiple studies were included in order to repeatedly replicate the main findings. Some results, if meant as illustrations, e.g. the circular feature of the boundary vector population in extended figure 1(b), were not pursued for replication, as it was not deemed necessary.
Randomization	The study did not involve any experimental subject groups; therefore, random allocation did not apply and was not performed.
Blinding	The study did not involve any experimental subject groups; therefore, experimenter blinding did not apply and was not performed.

Reporting for specific materials, systems and methods

We require information from authors about some types of materials, experimental systems and methods used in many studies. Here, indicate whether each material, system or method listed is relevant to your study. If you are not sure if a list item applies to your research, read the appropriate section before selecting a response.

Materials & experimental systems

n/a	Involved in the study
☒	☐ Antibodies
☒	☐ Eukaryotic cell lines
☒	☐ Palaeontology and archaeology
☒	☐ Animals and other organisms
☒	☐ Clinical data
☒	☐ Dual use research of concern
☒	☐ Plants

Methods

n/a	Involved in the study
☒	☐ ChIP-seq
☒	☐ Flow cytometry
☒	☐ MRI-based neuroimaging

Plants

Seed stocks	No plants were used in this study
Novel plant genotypes	N/A
Authentication	N/A
